# Neutrophil-to-Lymphocyte Ratio as a Predictor of Mortality for COVID-19-Related Acute Respiratory Distress Syndrome (ARDS) Patients Requiring Extracorporeal Membrane Oxygenation Therapy

**DOI:** 10.7759/cureus.46238

**Published:** 2023-09-29

**Authors:** Jeffrey Lu, Allison Karwoski, Lena Abdulrahman, Swati Chaparala, Mirnal Chaudhary, Khanjan Nagarsheth

**Affiliations:** 1 Vascular Surgery, University of Maryland School of Medicine, Baltimore, USA

**Keywords:** case cohort, critical care, lymphocytes, neutrophils, vv-ecmo, hypercoagulable state, acute respiratory distress syndrome, covid-19

## Abstract

Background: The neutrophil-to-lymphocyte ratio (NLR) has been studied as an indicator of systemic inflammation and as a prognostic tool in multiple areas of medicine. Previous research has suggested that higher NLR and rapid increase to peak NLR are associated with poorer outcomes in patients with coronavirus disease 2019 (COVID-19), particularly in those experiencing acute respiratory distress syndrome (ARDS). Within vascular surgery, there is data to suggest a positive correlation between elevated pre-extracorporeal membrane oxygenation (ECMO) NLR and higher rates of mortality following major procedures. This study explores the prognostic value of peri-ECMO NLR in patients requiring veno-venous ECMO (VV-ECMO) therapy for COVID-19-related ARDS. The objective of this study was to explore the utility of pre-ECMO NLR as an easily accessible prognostic factor for patients suffering from COVID-19-associated ARDS that require VV-ECMO.

Methods: This was a retrospective cohort study within a tertiary care hospital conducted between April 2020 and January 2021. Patients requiring VV-ECMO therapy for COVID-19-associated ARDS were included. Peri-ECMO NLR values, length of stay (LOS), duration on VV-ECMO, and discharge status were recorded. Receiver operating characteristic (ROC) curve analysis and Youden’s J statistics were performed to calculate a cut-off value of 11.005 for pre-ECMO NLR and 17.616 for on-ECMO NLR. Pre-ECMO and on-ECMO Kaplan-Meyer curves were generated for two groups of patients, those above and below NLR cutoff thresholds. Two-sample T-test was performed to test for significant differences in LOS and duration on VV-ECMO.

Results: Twenty-six patients were included in the study for final analyses. There was an overall mortality of 39% (n = 10). ROC curve analysis and Youden’s J statistic revealed an optimal cut-off value of pre-ECMO NLR = 11.005 and on-ECMO NLR = 17.616. Results showed that the patient group placed on VV-ECMO with a pre-ECMO NLR less than 11.005 experienced no mortality (n = 7) and a median LOS of 28 days (IQR = 14.5-64.5 days). The patient group on VV-ECMO with a pre-ECMO NLR greater than 11.005 (n = 19) included all mortality (n = 10) and had a median LOS of 49 days (IQR = 25.5-63.5 days). The patient group with on-ECMO NLR less than 17.616 also conferred a survival advantage. There was no significant difference in LOS or duration on VV-ECMO between the two groups, pre-ECMO or on-ECMO.

Conclusions: A pre-ECMO NLR cutoff was identified and offered statistically significant prognostic value in predicting mortality. A lower on-ECMO NLR value also indicated a survival advantage. Future studies should include NLR within multivariate models to better discern the effect of NLR and elucidate how it can be factored into clinical decision-making. Importantly, this data can be expanded to assess the predictive value of NLR pertaining to the COVID-19-induced ARDS population and matched cohorts.

## Introduction

Acute respiratory distress syndrome (ARDS) is a known complication of coronavirus disease 2019 (COVID-19) infection and has a significant impact on survival, having been associated with up to 10.8% of all COVID-19-related deaths [1,2]. It has been shown that the development of ARDS in COVID-19 patients is associated with a higher risk of mortality [3]. Early recognition of impending respiratory failure (saturation of peripheral oxygen (SpO2) <90% or respiratory rate (RR) >30bpm) and aggressive management of ARDS with a focus on limiting the inflammatory-mediated damage to lung epithelium in COVID-19 patients is crucial to improve outcomes and increase survival. Increased severity of ARDS, as measured by the ratio of partial pressure of oxygen to fraction of inspired oxygen (PaO2/FiO2), is strongly associated with decreased rates of survival in COVID-19 patients [4]. Patients with a lower PaO2/FiO2 ratio have been found to have a higher risk of mortality [5,6]. Consequently, the need for mechanical ventilation, extracorporeal membrane oxygenation (ECMO), and other advanced life support measures have also been associated with higher rates of pulmonary complications throughout hospital stay and higher mortality in patients with COVID-19-associated ARDS [7].

Veno-venous ECMO (VV-ECMO) is a form of advanced life support that can be used to help patients suffering from COVID-19-associated ARDS [7]. In contrast to veno-arterial ECMO (VA-ECMO), whose purpose is to restore blood flow and organ perfusion in the setting of cardiogenic shock or cardiac arrest, VV-ECMO provides respiratory support by oxygenating the blood while simultaneously removing carbon dioxide from the body. This therapy is considered a treatment option for critically ill patients who have failed conventional treatments such as mechanical ventilation and high-flow oxygen therapy. VV-ECMO initiation is well described in the literature and requires venous cannulation for connection to the ECMO circuit [8].

The effect of VV-ECMO on patient outcomes is influenced by various prognostic factors such as patient age, co-morbidities, severity of illness, and duration of ECMO therapy [9,10]. Additionally, factors related to the cannulation procedure itself, such as cannulation site, type, and timing, have also been shown to influence outcomes [11,12]. Recently, studies have investigated the use of the absolute neutrophil count to absolute lymphocyte count ratio or neutrophil-to-lymphocyte ratio (NLR) as an indicator of clinical course for patients suffering from ARDS [13,14]. This study builds upon that knowledge by investigating the use of peri-ECMO NLR values as an easily accessible biomarker of prognosis for patients on VV-ECMO for ARDS secondary to COVID-19.

## Materials and methods

Study setting

This study was conducted in an academic tertiary care center with approximately 220 critical care beds serving as a statewide referral center (University of Maryland Medical Center, Baltimore, Maryland, United States). A separate intensive care unit (ICU) with 32 beds and a dedicated medical team was established to care for patients on VV-ECMO. This study included all patients in the unit who were receiving treatment after a confirmed diagnosis of COVID-19-related ARDS. This study was approved by the University of Maryland School of Medicine Human Research Protections Office (approval number: HP-00100056).

Patient selection and study design

We conducted a retrospective review of all patients who underwent VV-ECMO cannulation at our institution for COVID-19-related ARDS between April 2020 and January 2021. Inclusion criteria were: patients who were at least 18 years old and cannulated for VV-ECMO with COVID-19 ARDS. COVID-19 was diagnosed based on a positive polymerase chain reaction (PCR) test of nasal or pharyngeal swab specimens in all patients. All patients were cannulated using a 25 French right internal jugular vein catheter and a 23 French right femoral vein catheter. After cannulation, all patients were maintained on intravenous heparin for a target-activated partial thromboplastin time (aPTT) of 45-55 seconds. In the event of significant bleeding episodes, heparin administration was withheld until bleeding complications resolved or lab results demonstrated stable hemoglobin and hematocrit values. If heparin-induced thrombocytopenia (HIT) occurred, a second-line anticoagulant, bivalirudin, was administered with a target aPTT goal of 46-76 seconds. All patients were cannulated for a continuous period without de-cannulation at any point until recovery or expiration. All VV-ECMO data regarding anticoagulation goals were documented in a separate note in the electronic health record system (EHR).

Data collection

Using a standardized data form, data was abstracted by research staff from the EHR. Abstractors were blinded to the study hypothesis. After training in data collection by the principal investigator, the charts were double-checked randomly for reliability by one of the investigators to maintain inter-rater reliability. Clinical data collected for these patients included baseline co-morbidities, demographic information, anticoagulation, pertinent pharmacotherapy, laboratory test results throughout their hospital stay, bleeding and thrombotic complications, and disposition (Table 1). Routine blood examinations consisted of a differentiated complete blood count and coagulation profile. The independent variable under investigation was the NLR. Primary outcomes that were investigated include length of stay (LOS) and disposition, with expiration and discharge being the possible outcomes. All laboratory data from the date of admission to the date of discharge were recorded.

**Table 1:** Demographic Data of Patient Population GERD: gastroesophageal reflux disease; CKD: chronic kidney disease

Demographic		Number of patients (n=26)	Percentage
Age (years)	20-29	3	12%
	30-39	5	19%
	40-49	9	35%
	50-59	9	35%
	Average (± SD)	44.5 (± 10.5) years	
	Range	26-58 years	
Gender	Female	7	27%
	Male	19	73%
Race	White	2	8%
	Black	7	27%
	Asian	1	4%
	Hispanic	14	54%
	Other	2	8%
Anti-Coagulation	Heparin/Enoxaparin	23	88%
	Xa Inhibitor	2	8%
	Direct thrombin Inhibitor	1	4%
Pharmacotherapy	Anti-virals	12	46%
	Steroids	22	85%
Baseline Co-Morbidities	Diabetes	7	27%
	Hypertension	9	35%
	Dyslipidemia	3	12%
	Hypothyroidism	3	12%
	Asthma	1	4%
	GERD	2	8%
	CKD	2	8%
	Cardiovascular disease	2	8%
Overall Mortality Rate		10	38%

Statistical analysis

Primary statistical analysis of the dataset began with the dichotomization of NLR values into Pre-ECMO and On-ECMO subsets. Pre-ECMO NLR values were calculated for each patient by averaging NLR values within four days prior to ECMO cannulation. On-ECMO NLR values were calculated for each patient by averaging NLR values after cannulation throughout the remainder of the patients’ hospital stay. Next, logistical regression and receiver operating characteristic (ROC) curve analysis were performed for the Pre-ECMO and On-ECMO NLR subsets. Confusion matrices based on sensitivity and specificity for expiration were formulated for each potential NLR cutoff value, and Youden’s J statistic (p<0.05) was calculated to determine the optimal Pre-ECMO and On-ECMO NLR cutoff values. 

After calculating the Pre-ECMO NLR cutoff value, the patient cohort was organized into two groups: those who were above the cutoff (high-risk) and those who were underneath the cutoff (low-risk). This step was also repeated for the On-ECMO subset as well. Statistical significance in outcome was assessed through multivariate linear regression analysis with an emphasis on maximizing correlation. In addition, non-parametric Kaplan-Meier curves were generated to assess for pre-ECMO and on-ECMO differences in mortality between the two groups as a function of duration on VV-ECMO. Differences in survival probability between the two dichotomized groups were assessed using the log-rank test for Kaplan-Meier analysis (p<0.05) [12]. In addition, a two-sample T-test was performed to examine differences in length of stay (LOS) and duration of VV-ECMO.

## Results

A total of 26 patients were included in the study for final analyses. Patients on ECMO demonstrated an overall mortality of 39% (n = 10). The average LOS to discharge among all patients was 59 days with a median of 55 days (IQR = 30.75-80.75 days). The average time on VV-ECMO among all patients was 48.4 days with a median of 47 days (IQR = 20.75-63.75 days). Twelve patients (46%) received anti-viral pharmacotherapy and 22 patients (85%) received steroids. The average NLR value across the entire patient cohort for the pre-ECMO time period was 20.98. The average for the on-ECMO time period was 17.18. The overall average daily NLR value across all patients was 16.636 (Table 2). A total of 23 patients were administered heparin or enoxaparin for anti-coagulation (88%), two were on a factor Xa inhibitor (8%), and one was on a direct thrombin inhibitor (4%). Of these patients, a total of 14 patients developed thrombotic complications (54%) and 15 patients developed bleeding complications (58%). Of the 14 patients, 13 survived the adverse thrombotic events (93%) and eight out of 15 patients survived the adverse bleeding events (53%) (Table 3). In the high-risk pre-ECMO patient group, nine patients (48%) developed thrombotic complications and 11 patients (58%) developed bleeding complications. In the high-risk on-ECMO patient group, one patient (11%) developed thrombotic complications and seven (78%) developed bleeding complications (Table 4).

**Table 2:** Average NLR Values of Patient Cohort NLR: neutrophil-to-lymphocyte ratio; ECMO: extracorporeal membrane oxygenation

NLR Values	ECMO Status	Patient Average	Calculated Threshold
	Pre-ECMO	20.98	11.005
	On-ECMO	17.18	17.616
	Overall Average NLR	16.636	

**Table 3:** Rates of Thrombotic and Bleeding Complications

	Total Number of Patients	Number that Survived	Survival Rate
Thrombotic Complications	14	13	93%
Bleeding Complications	15	8	53%

**Table 4:** Rates of Thrombotic and Bleeding Complications in the High-Risk Population ECMO: extracorporeal membrane oxygenation

	Number of Patients above Cut-Off	Thrombotic Complications	Bleeding Complications
Pre-ECMO	19	9 (~48%)	11 (~58%)
On-ECMO	9	1 (~11%)	7(~78%)

**Figure 1:**
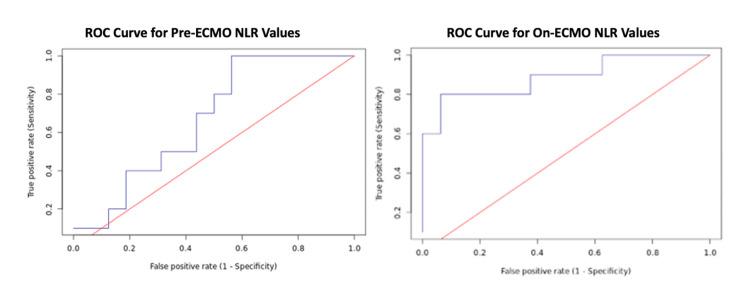
ROC Curves Left: Youden’s J Statistic NLR Cutoff = 11.005; Area Under Curve = 0.70 Right: Youden’s J Statistic NLR Cutoff = 17.616; Area Under Curve = 0.89 NLR: neutrophil-to-lymphocyte ratio; ROC: receiver operating characteristic; ECMO: extracorporeal membrane oxygenation

**Figure 2:**
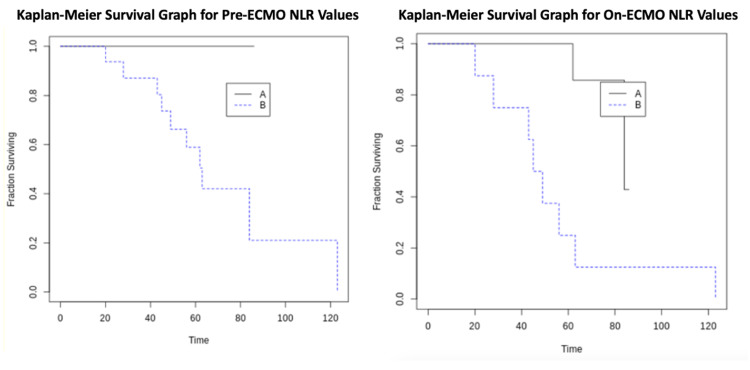
Kaplan-Meier Curves Left: NLR Cutoff = 11.005; A = Patients NLR < 11.005; B = Patients NLR > 11.005 Right: NLR Cutoff = 17.616; A = Patients NLR < 17.616; B = Patients NLR > 17.616 NLR: neutrophil-to-lymphocyte ratio; ECMO: extracorporeal membrane oxygenation

Pre-ECMO, ROC curve analysis and Youden’s J statistic revealed an optimal pre-ECMO NLR value of 11.005 (sensitivity = 1; specificity = 0.438). On-ECMO, ROC curve analysis and Youden’s J statistic also revealed an optimal averaged on-ECMO NLR value of 17.616 (sensitivity = 0.8; specificity = 0.938) (Figure 1). Kaplan-Meier curves showed that the patient group placed on VV-ECMO with lower pre-ECMO NLRs (less than 11.005) experienced no mortality (n = 7) while the patient group with higher pre-ECMO NLRs (greater than 11.005; n = 19) included all mortality (n = 10). On-ECMO, Kaplan-Meier for lower NLRs (less than 17.616; n=17) experienced a mortality rate of 12% (n = 2) while the patient group with averaged higher on-ECMO NLRs (greater than 17.616; n = 9) experienced a mortality of 89% (n = 8) (Figure 2). Multivariate linear regression revealed a statistically significant difference in survival between those who had higher and lower pre-ECMO NLR values (p = 0.05). On-ECMO, multivariate linear regression also revealed a statistically significant difference in survival between those who had higher and lower on-ECMO NLR values (p = 0.007) (Table 5). Multivariate regression analysis also demonstrated a strong correlation between the calculated thresholds and the effect on disposition (r = 0.886). Although there did not seem to be a definitive survival advantage between the two pre-ECMO groups, Kaplan Meier log-rank test did show that it was approaching significance (p = 0.097). On-ECMO, Kaplan Meier log-rank test showed that there was a significant survival advantage for the patient group with lower on-ECMO NLRs (p = 0.0028) (Table 6).

**Table 5:** Multi-Variate Linear Regression with Disposition as the Dependent Variable NLR: neutrophil-to-lymphocyte ratio; ECMO: extracorporeal membrane oxygenation

Predictor	Estimate	Standard Error	t statistic	p-value
Intercept	-0.03109	0.29848	-0.1042	0.918
Pre-ECMO NLR Threshold	0.28177	0.13527	2.0830	0.053
On-ECMO NLR Threshold	0.50772	0.16660	3.0476	0.007
Length on-ECMO (days)	0.00224	0.00254	0.8835	0.389
Gender	0.16432	0.14588	1.1264	0.276
Race/Ethnicity				
Hispanic-White	-0.20024	0.23340	-0.8579	0.403
Black-White	-0.37106	0.26913	-1.3787	0.186
Asian-White	-0.03392	0.44295	-0.0766	0.940
Other-White	0.18890	0.29427	0.6419	0.529
Model Fit & Correlation				
R		0.886		
R^2^		0.784		

**Table 6:** Kaplan-Meier Rank Log Test NLR: neutrophil-to-lymphocyte ratio; ECMO: extracorporeal membrane oxygenation

Log-Rank Test Parameter	Pre-ECMO NLR Threshold	On-ECMO NLR Threshold
Chi-Square Statistic	2.758	8.937
Deg. of Freedom	1	1
p-value	0.097	0.0028

Regarding LOS, the patient group with pre-ECMO NLRs less than 11.005 had a median LOS of 38 days (interquartile range (IQR) = 28.5-86.5 days) and median time on VV-ECMO of 28 days (IQR = 14.5-64.5 days). The patient group with pre-ECMO NLRs greater than 11.005 had a median LOS of 59 days (IQR = 38-68.5 days) and a median time on VV-ECMO of 49 days (IQR = 25.5-63.5 days). There was no significant difference in LOS (p = 0.449) or time on VV-ECMO (p = 0.196) between the two groups. The patient group with average on-ECMO NLRs less than 17.616 had a median LOS of 66 days (IQR = 33-84 days) and a median time on VV-ECMO of 58 days (IQR = 18-71 days). The patient group with on-ECMO NLRs greater than 17.616 had a median LOS of 50 days (IQR = 30-59 days) and a median time on VV-ECMO of 45 days (IQR = 28-56 days). There was no significant difference in LOS (p = 0.186) or time on VV-ECMO (p = 0.472) between the two groups.

## Discussion

More research is warranted to determine the optimal values for severe inflammatory pathologies, such as ARDS or sepsis, since NLR has predominantly been studied in the field of oncology. In general, it has been shown that ratios of approximately 3, 6, and 9 indicate states of being healthy, mildly ill, and severely ill, respectively [15,16]. In terms of COVID-19, studies have shown that an NLR > 5 correlates to an elevation of other biochemical markers and indicates more severe infection [17]. Given this study’s overall average NLR value of 16.636, patients with COVID-19-associated ARDS can be classified as critically ill. Studies have shown that our NLR values may be elevated due to the hypercoagulable state induced by COVID-19 infection, skewing complete blood count (CBC) parameters [18]. Nevertheless, aggressive management through modalities such as VV-ECMO is considered for these patients for survival. Lately, there has been discussion regarding the use of NLR in determining candidacy for surgical intervention for malignancy. Studies have shown that a pre-op NLR >5 is associated with decreased post-op survival for patients with metastatic colorectal cancer [19]. However, due to the COVID-19 pandemic, more recent data has emerged investigating NLR in the context of cardiovascular intervention. For example, Appleton et al. reported that in a study of 350 patients undergoing open abdominal aortic aneurysm (AAA) repair, all deaths at 30-day with a pre-op NLR >5 were a result of myocardial infarction and confirmed by autopsy and death certification data [20]. Although mechanisms are still poorly understood, it is hypothesized that chronic inflammation with increased neutrophil infiltration and extravasation not only progresses but also disrupts atherosclerotic plaque, which can cause an increase in the incidence of major cardiovascular events [21]. In terms of peripheral vascular disease, Luo et al. have shown that rates of amputation-free survival are higher in patients with critical limb ischemia if NLR <3.8 after medical management than in patients with NLR >3.8 [22]. Regardless, these NLR values seem to be significantly lower than our calculated NLR values of pre-ECMO NLR = 11.005 and on-ECMO NLR = 17.616.

The use of VV-ECMO for ARDS has significantly evolved over the years, becoming a valuable therapeutic option that decreases morality in certain critical care settings [23]. According to the Extracorporeal Life Support Organization (ELSO), current criteria warranting cannulation include hypoxemic respiratory failure (PaO2/FiO2 < 80 mm Hg), hypercapnic respiratory failure (pH < 7.25; RR = 35; plateau pressure <30 cmH2O), or ventilatory support as a bridge to lung transplantation [24]. In the setting of critical illness, VV-ECMO has predominantly served as a rescue therapy when conventional strategies, such as prone positioning and mechanical ventilation, fail to adequately support patients with severe ARDS. However, there is conflicting literature regarding whether VV-ECMO cannulation truly offers mortality benefits in this patient population. Based on the ECMO to Rescue Lung Injury in Severe ARDS (EOLIA) trial, the overall use of VV-ECMO for ARDS does not seem to offer significant mortality benefits [25]. However, it is worth noting that this trial was also stopped early for interim results due to suggested futility with initial results demonstrating an 11% absolute difference in 60-day mortality. In terms of COVID-19-related ARDS, no definitive criteria were ever established as official guidelines for VV-ECMO cannulation. Thus, clinicians often referred to the original guidelines established by ELSO for patient management throughout the pandemic. Preliminary data seems to support the use of VV-ECMO for refractory hypoxemia due to COVID-19-related ARDS, with Bertini et al. citing a mortality rate of ~39% (vs. 46% in the EOLIA trial), however, more research is needed to draw a definitive conclusion [26,27]. In addition, studies have shown that factors such as age, severity of hypoxemia, and duration/intensity of mechanical ventilation can be significant modifiers of treatment effectiveness in the COVID-19-related ARDS patient population [28].

It is also important to consider the clinical implications of ARDS that is associated with COVID-19. ELSO indicates that having no transition to a defined endpoint (“bridge to nowhere”) and coagulopathies are significant contraindications to VV-ECMO cannulation. The underlying philosophy is that without a clear transition plan to determine when to wean off ECMO support, patients are subjected to prolonged treatment with further potential complications that can outweigh the benefits. Alternatively, patients with coagulopathies are another significant concern as ECMO circuits can activate the body's coagulation cascade and lead to life-threatening complications such as hematomas or thrombosis within the circuit.

With COVID-19 being well cited in the literature to induce cytokine storms, patients with COVID-19-related ARDS are highly susceptible to hyperinflammatory responses that can significantly worsen respiratory status. Thus, the physiological reaction to such cytokine storms can exacerbate coagulopathy, making it even more challenging to manage patients on ECMO due to the increased risk of thrombotic and bleeding complications. With NLR being a known indicator of inflammatory status, multiple studies have shown that higher NLR values are associated with an increased risk of bleeding and thrombotic complications [29,30]. As a result, COVID-19 patients with ARDS may present unique challenges in ECMO selection, and careful consideration of their inflammatory status and coagulation profile is crucial to optimizing outcomes and reducing the risks associated with VV-ECMO support in this population. In addition, there is ongoing debate in the medical community regarding the optimal timing for VV-ECMO cannulation in patients suffering from COVID-19-related ARDS. Some suggest that earlier cannulation can help to prevent the progression of ARDS and reduce the need for more advanced life support measures, thus reducing the patient's risk of death and improving their outcome [31,32]. On the other hand, others argue that early cannulation may not be necessary in all cases and that it should be reserved for patients with the most severe forms of ARDS since it is considered a last-line therapy. Although the decision of when to cannulate for VV-ECMO in these patients should be made on a case-by-case basis, NLR values could serve as a useful indicator for guiding these clinical decisions and could also prove to be a reliable marker for on-ECMO monitoring.

In the context of ECMO, where patients are subject to a pro-inflammatory state and a heightened risk of deep vein thrombosis (DVT) and other thrombotic/bleeding complications, monitoring NLR could offer valuable insights. By regularly measuring NLR levels in ECMO patients, clinicians may be able to identify those at higher risk of developing DVT and tailor preventive strategies accordingly. This could include more vigilant monitoring, early initiation of prophylactic anticoagulation prior to ECMO, or adjustments to the anticoagulation regimen during ECMO support. However, it is crucial to recognize that NLR is just one of several factors contributing to DVT risk in ECMO patients, and its use in clinical decision-making should be combined with other established risk assessment tools and thorough clinical evaluation. Further prospective studies are warranted to validate the utility of NLR in managing DVT risk in ECMO patients with COVID-related ARDS and to determine its role in optimizing patient outcomes.

Limitations

This study explores the prognostic significance of NLR in patients undergoing ECMO at a single center. While the findings contribute valuable insights, several limitations should be acknowledged to provide a comprehensive understanding of the study's results. One of the primary limitations of this study is the relatively small sample size of patients included. The limited sample size may restrict the generalizability of the findings to a broader population. Although the study aimed to ensure rigor in data collection and analysis, the small sample size might compromise statistical power and hinder the ability to detect more subtle associations or effects. In addition, the absence of a comparison group with NLR values surrounding alternative treatment modalities restricts the ability to assess the unique utility that NLR might offer in patients suffering from ARDS secondary to COVID-19. A comparative analysis between the two groups could have elucidated potential differences in clinical characteristics or disease trends, offering a more robust understanding of the role of NLR. Furthermore, the lack of a standardized understanding of NLR can also profoundly impact the interpretation of results. This case series relied upon the analysis of patient NLR values to identify patterns, associations, and outcomes in the prognosis of patients on VV-ECMO for ARDS secondary to COVID-19. Although NLR can be a potential indicator of systemic inflammation and offer valuable insights into disease progression and therapeutic responses, the absence of established reference ranges and a clear grasp of how NLR values fluctuate in diverse clinical scenarios and patient populations make the interpretation of findings somewhat difficult. This lack of standardization can limit the ability of clinicians to draw meaningful conclusions from this data, hampering the capacity to comprehend the clinical significance of NLR-related trends in real-world healthcare settings.

Several patients included in this study originally presented at various outside institutions with varying baseline health characteristics and co-morbidities, adding an additional layer of complexity that obscures the interpretation of the data. NLR is influenced by various factors beyond the scope of this study, such as age, co-morbidities, and underlying health conditions. For example, the clinical use of steroids and remdesivir has been known to decrease NLR values [33]. Despite the use of this pharmacotherapy, the NLR values within our study still remained significantly higher than what has been reported, questioning the role of these medications and their effect on NLR values. Although efforts were made to control for known confounders during the statistical analysis, the presence of unaccounted confounding variables could limit the study's conclusions. Lastly, ECMO cannulation criteria during the COVID-19 pandemic might vary according to specific site practices, protocols, and expertise at single centers. Differences in patient management, care protocols, and ECMO implementation across different centers may affect patient outcomes and the association.

## Conclusions

The outcomes of VV-ECMO cannulation for COVID-19-associated ARDS are influenced by a variety of factors. However, peri-ECMO NLR values could prove to be a reliable and easily accessible prognostic indicator of clinical course for such patients. In this study, pre-ECMO and on-ECMO NLR cutoffs were identified and offered statistically significant prognostic value in predicting mortality. Our pre-ECMO NLR data showed a difference in mortality that was approaching significance, which demonstrates that patients with a higher pre-ECMO NLR had a significantly higher risk of complications, morbidity, and mortality when compared to those with a lower pre-ECMO NLR. A lower on-ECMO NLR value seemed to indicate a survival advantage. Specifically, the on-ECMO NLR data found in this study seems to suggest that patients with a lower on-ECMO NLR are more responsive to treatment and experience better prognosis when compared to those with a higher on-ECMO NLR. Future studies should include NLR within multivariate models to better discern the effect of NLR and elucidate how it can be factored into clinical decision-making. Importantly, this data can be expanded to assess the predictive value of NLR pertaining to the COVID-19-induced-ARDS population and matched cohorts. Additional research on the use of peri-operative NLR as a prognostic indicator for VV-ECMO cannulation can prove to be valuable in determining management plans for critically ill patients.
